# Transmembrane Protein-Based Risk Model and H3K4me3 Modification Characteristics in Lung Adenocarcinoma

**DOI:** 10.3389/fonc.2022.828814

**Published:** 2022-03-22

**Authors:** Tao Fan, Yu Liu, Hengchang Liu, Liyu Wang, He Tian, Yujia Zheng, Bo Zheng, Liyan Xue, Chunxiang Li, Jie He

**Affiliations:** ^1^ Department of Oncology, Renmin Hospital of Wuhan University, Wuhan, China; ^2^ Department of Thoracic Surgery, National Cancer Center/National Clinical Research Center for Cancer/Cancer Hospital, Chinese Academy of Medical Sciences and Peking Union Medical College, Beijing, China; ^3^ Department of Colorectal Surgery, National Cancer Center/National Clinical Research Center for Cancer/Cancer Hospital, Chinese Academy of Medical Sciences and Peking Union Medical College, Beijing, China; ^4^ Department of Pathology, National Cancer Center/National Clinical Research Center for Cancer/Cancer Hospital, Chinese Academy of Medical Sciences and Peking Union Medical College, Beijing, China

**Keywords:** transmembrane proteins (TMEMs), tumor microenvironment (TME), H3K4me3 modification, immune checkpoint inhibitor (ICI), gene mutation

## Abstract

The role and mechanism of transmembrane proteins (TMEMs) in tumorigenesis remain unclear. Based on 4 independent cohorts containing 1,208 cases, we identified 3 TMEMs (TMEM273, TMEM164, and TMEM125), which were used to construct a risk model to predict the prognosis of LUAD. The two patterns based on the risk score exhibited a high degree of consistency with the characteristics of immune cell infiltration and epigenetic distribution. Patients with a low-risk score, characterized by an increased activation of immunity, H3K4me3 modification, tumor cell apoptosis, chemokine secretion, and TMB, had better disease-free survival (DFS) and overall survival (OS). Obvious immunosuppression, increased epithelial–mesenchymal transition, a low H3K4me3 level, shortened cell cycle, and accelerated cell division manifested in high-risk patients, with poorer DFS and OS. The model showed a better prognostic value than the tumor immune dysfunction and exclusion score. Correlation analysis told us that patients with high scores were suitable for treatment with CD276 inhibitors for their higher levels of CD276 expression. The risk score had a strong negative correlation with HAVCR2 and ICOS among patients with EGFR-WT, KRAS-WT, STK11-WT, or TP53-MUT, and patients with these mutation types with low scores were suitable for treatment with HAVCR2 or ICOS inhibitors. This work comprehensively analyzed the role and mechanism of TMEMs in LUAD and revealed the characteristics of histone methylation modification. The TMEM-based signature gave us deep insight into immune cell infiltration profiles and provided an individualized immunotherapy strategy.

## Introduction

A transmembrane protein (TMEM), also known as an integral membrane protein, is the most important component of membrane proteins. Membrane proteins classified as TMEM must be embedded in or span at least one segment of the biological membranes (1). In view of the structure and distribution characteristics of the TMEM, it exhibits unique biological functions. The cooperation of TMEM242 and TMEM70 with mitochondrial complex I assembly (MCIA) was involved in the assembly of ATP synthase and played an important role in ATP energy metabolism (2). TMEM106C was overexpressed in hepatocellular carcinoma (HCC), and the inhibition of TMEM106C in liver cancer cell lines using small interfering RNA significantly suppressed the cell proliferation, migration, and invasion ability (3). In addition, TMEM regulated the tumor progress by the modulation of immune response. The well-known TMEM173, also referenced as the stimulator of interferon genes (STING), has been found to play an important role in anti-viral innate immunity and anti-tumor immunity (4–8). Increasing studies indicated that the abnormal expression of TMEM was closely related to tumor occurrence, progression, and metastasis and immunomodulatory abnormality.

The immune status in the tumor microenvironment (TME) determined the patient’s prognosis. The release of chemokines, chemotaxis of macrophages, antigen presentation, and function of immune surveillance are all key aspects of the body’s immunity to tumors (9–11). The latest tumor immunity research has found that some special molecules were expressed on the surface of immune cells in the TME. Instigated by tumor cells, they could prevent immune cells from activating and inhibit immune cells from attacking tumor cells. Such molecules are called immune checkpoints, and their discoveries bring hope to humans to overcome or cure tumors. At present, more than 10 immune checkpoint molecules [CD274(PD-L1),CD276, CTLA4, HAVCR2, ICOS, IDO1, LAG3, PDCD1(PD1), et al.] were discovered to have amazing tumor treatment effects in some patients. However, most patients still do not have a good effect, which is far from meeting clinical needs (12). In the treatment of lung adenocarcinoma (LUAD), immune checkpoint inhibitor (ICI) therapy still has excellent performance. Compared with patients with a low expression of PD-L1, NSCLCs with a PD-L1 expression of ≥90% exhibited significantly improved clinical outcomes (13). LUAD patients with a co-expression of PD-L1 and IDO1 was significantly associated with poor OS and disease-free survival (14). Current clinical trial research showed that the IDO1 enzyme inhibitor had encouraging antitumor activity in multiple advanced solid tumors (15). Although a variety of ICIs have entered the clinical or pre-clinical stage, the choice of people who benefit from different ICIs is still the key to improve the cure rate of tumors. For example, the expression of PD-L1 in patients with LUAD was significantly associated with the EGFR mutation status and KRAS mutation status, which meant that patients with different mutation types responded differently to anti-PD-L1 treatment (16). Patients with acute myeloid leukemia (AML) with TP53 mutation often have a poor prognosis. Compared with wild-type AML, patients with TP53 mutation displayed significantly decreased ICOS, which may be the primary factor leading to poor outcomes (17). In view of this, there is an urgent need to establish more methods to screen specific populations suitable for treatment with different ICIs.

According to reports, the role of epigenetic modification in tumor immunity is increasingly becoming important (18, 19). The trimethylation of histone 3 lysine 4 (H3K4me3) modification was widely found in regulating the gene transcription, cell cycle, apoptosis, and tumor immunity (20–22). Macrophages showed a marked global enrichment of H3K4me3 after being stimulated by immune complexes and lipopolysaccharide (LPS) (23). All these studies are about the regulatory relationship between individual genes and histone trimethylation. They did not comprehensively analyze the level of histone trimethylation in cancer patients from the level of whole-genome sequencing, nor did they explore the effect of histone trimethylation level on the prognosis of patients from the clinical cohort. Of course, there is no research on the regulatory relationship between a specific molecular family and histone trimethylation.

In recent years, with the development of multi-omics, the analysis based on whole-genome expression data provides a new method for the screening of prognostic indicators for cancer patients. Especially in the field of LUAD research, many biometric analyses based on sequencing data have found results that can better predict tumor prognosis and tumor immunotherapy response (24–26). However, these studies have integrated the expression data of all genes and have not conducted in-depth studies on specific molecular families; there may be biases when performing functional analysis, or the analysis may not fully reflect the biological characteristics of tumors.

For the first time, we performed a comprehensive analysis of the expression characteristics of TMEMs in patients with LUAD and its correlation with the clinical prognosis of patients. We constructed a signature based on 3 TMEMs (TMEM125, TMEM164, and TMEM273) using 477 LUAD cases collected from The Cancer Genome Atlas (TCGA) database, and the features and functions of this signature were verified by 3 other independent cohorts containing 731 LUAD cases. In addition, we identified special immune cell infiltration profiles and signal pathway enrichment characteristics related to TMEMs. More importantly, we discovered the H3K4me3 modification characteristics of 1,208 patients with LUAD and found that the level of histone modification was closely related to this TMEM-based signature, which played a key role in the progression of LUAD patients. Our research can help scholars to better understand the signature of the immune microenvironment and epigenetic modification characteristics of LUAD related to TMEMs and help clinicians to optimize tumor immunotherapy.

## Materials and Methods

### LUAD Data Collecting and Preprocessing

A total of 1,208 cases collected from 4 independent cohorts were included in this study. A total of 477 LUAD samples with full clinical annotation downloaded from TCGA (https://portal.gdc.cancer.gov/) were served as a training set. The gene expression data of other 731 LUAD samples (85 cases in GSE30219, 226 cases in GSE31210, and 420 cases in GSE72094) with overall survival (OS) or recurrence-free survival (RFS) information acquired from the GEO (https://www.ncbi.nlm.nih.gov/geo/) database were used as validation sets.

### Generation of Differentially Expressed TMEMs and Signature Construction

A total of 249 well-defined TMEM family genes were included for the difference analysis between 54 normal tissues and 497 tumor tissues based on the TCGA LUAD gene expression matrix. R package limma was used for gene difference analysis. |Log fold change (FC)|>1 and false discovery rate (FDR) <0.05 were served as the thresholds. Then, the differentially expressed genes (DEGs) were used for univariate Cox proportional hazards regression analysis, least absolute shrinkage and selection operator (LASSO) regression analysis with one standard error (SE) and 100-fold cross-validation, and multivariate Cox proportional hazards regression analysis, and obtained independent factors that affect the prognosis of the disease. This risk model was constructed based on the expression level and weight coefficient of the final selected genes. The risk score calculation formula is as follows:


$$risk\;score=\sum_{k=1}^{n}coef(k)*gene(k)$$

According to the risk score, patients were divided into the high-risk group and low-risk group.

### Biological Pathway and Functional Annotation

A correlation analysis between risk score and all genes were performed to screen out this signature-related genes. We performed the Kyoto Encyclopedia of Genes and Genomes (KEGG) and Gene Ontology (GO) enrichment analyses of these signature-related genes.

In order to better study the characteristics of signal pathways in the TME of patients in the high-risk group and low-risk group, gene set enrichment analysis (GSEA) and gene set variation analysis (GSVA) were performed using the R package of “org.Hs.eg.db,” “enrichplot,” and “GSVA.” For running GSVA analysis, the gene sets of “msigdb.v7.2.symbols” were acquired from the MSigDB database (http://www.gsea-msigdb.org/gsea/msigdb/). The functional annotation was completely implemented by R package of “clusterProfiler.”

### Estimation of Immune Cell Infiltration

The relative abundance of each type of immune cell was calculated by the algorithm of CIBERSORT R script v1.03 (27). CIBERSORT is widely used to calculate the proportion of immune cell infiltration in the TME of solid tumors (28–31).

### Tumor Mutation Burden and Tumor Immune Dysfunction and Exclusion Analysis

The TMB of patients with LUAD in the training set was downloaded from the website of https://portal.gdc.cancer.gov/. TIDE is a computational method to simulate the mechanism of tumor immune escape, which was firstly reported by Jiang and his colleagues in 2018 (32). The TIDE signature mainly contained T-cell exclusion and T-cell dysfunction, and their scores have been used to predict the ICIs’ clinical response and prognosis of NSCLC and melanoma (32–34). We could get the TIDE score, interferon gamma (IFNG) score, dysfunction score, exclusion score, and CD8 score from the website of http://tide.dfci.harvard.edu after uploading standardized transcriptome data.

### Survival Meta-Analysis and Statistical Analysis

The R package of “meta” was used to perform OS meta-analysis for the 4 independent cohorts (TCGA-LUAD, GSE30219, GSE31210, and GSE72094) and RFS meta-analysis for the 3 independent datasets (TCGA-LUAD, GSE30219, and GSE31210). The correlation analysis between the risk score and gene expression or gene set score were carried out using the R package of “limma” by Spearman. The patients in each cohort were divided into high- and low-risk groups according to their ranked scores. Kaplan–Meier curves were used to describe the OS and RFS of patients with LUAD in each cohort. The log-rank (Mantel–Cox) test was used to compare the difference in the OS and RFS between the high-risk group and the low-risk group. Univariate Cox regression analysis and LASSO regression analysis were used to reduce variables, and multivariate Cox regression analysis was used to screen out independent prognostic factors. The receiver operating characteristic (ROC) curves were used to evaluate the specificity and sensitivity of the risk score, TIDE score, IFNG score, dysfunction score, exclusion score, and CD8 score, and the R package of “timeROC” was used to quantify the area under the curve (AUC). A comparison of the immune score, stromal score, ESTIMATE score, immune cell infiltration abundance, and TMB between the high-risk group and the low-risk group was performed using *Graghad Prism 8.0.1* and Mann– Whitney *U*-test. *P*<0.05 was considered statistically significant. Data analysis and figure generation were done by R version 3.6.1.

## Results

### Construction of TMEM-Based Signature for LUAD Patients

**Figure 1:**
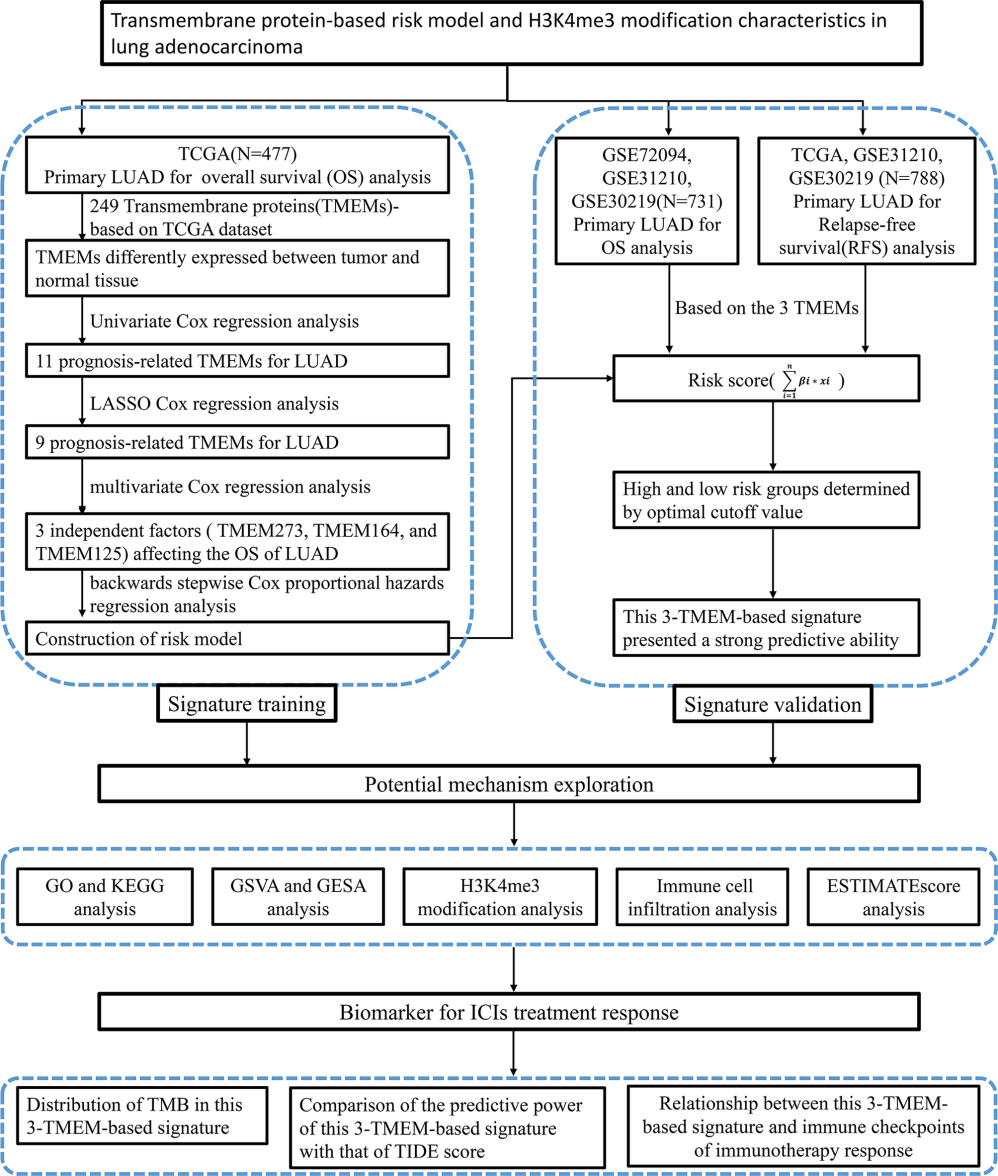
Flow chart of this research.

The screening process and research framework are shown in **Figure 1**. A total of 249 well-defined TMEM family genes were enrolled in this study. Based on the gene expression matrix of 54 normal tissues and 497 tumor tissues, 61 DEGs were identified according to the calculation standard of FDR < 0.05 and |logFC|>1 (**Figure S1A** and **Table S1**). A further univariate Cox regression analysis was performed to screen out 11 genes related to the OS of patients with LUAD (**Table S2**). In order to make the model more stable and easier to implement, LASSO regression analysis with one SE and 100-fold cross-validation was used to further filter the variables (**Figures S1B, C**). As a result, 9 genes were generated to be used for a multivariate Cox regression analysis (**Figure S1D**). At last, TMEM273, TMEM164, and TMEM125 were identified as the most important variables, which were independent factors affecting the prognosis of LUAD (**Figure S1D**).

### Landscape of the TMEM-Based Signature and Its Prognostic Value in LUAD

**Figure 2:**
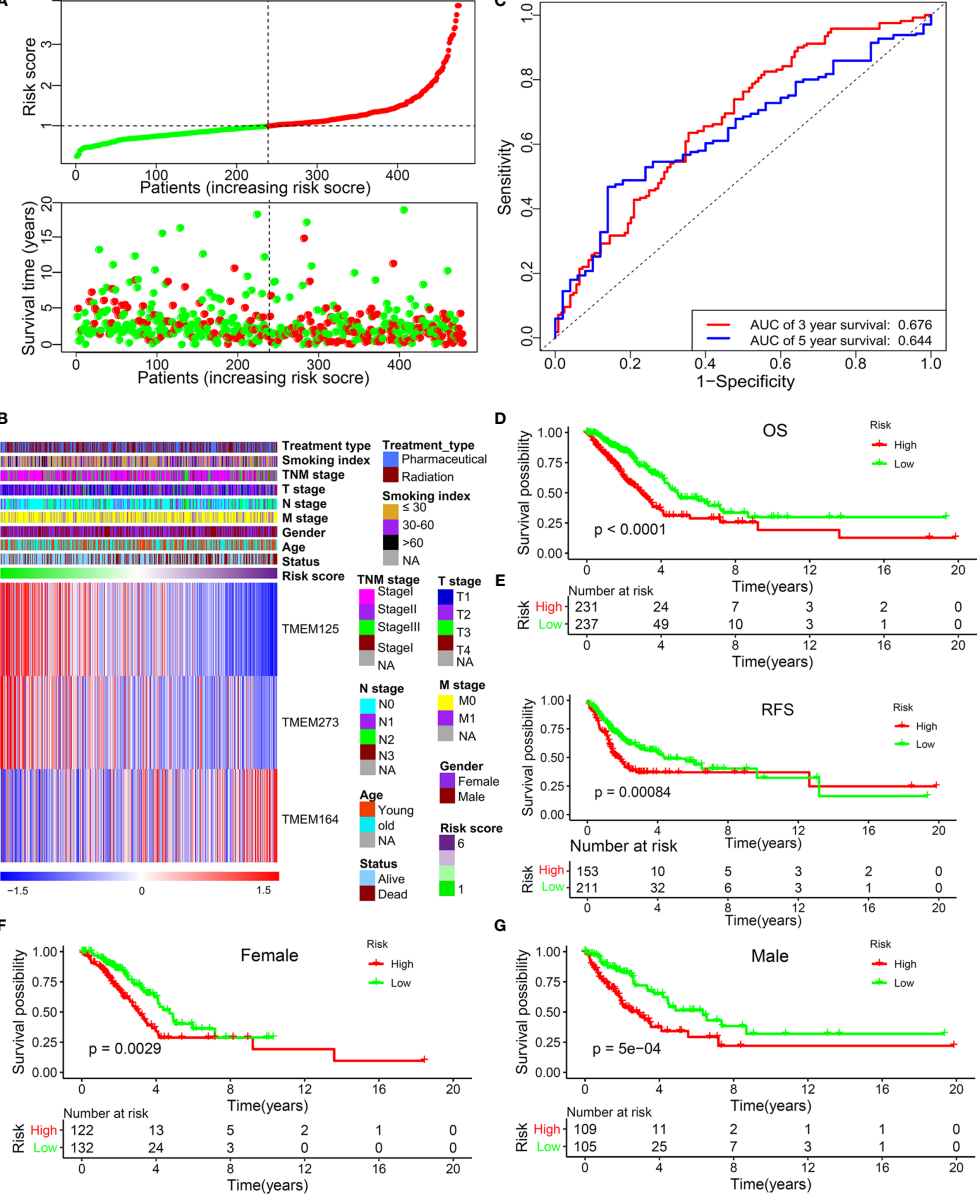
The predictive value of this TMEM-based signature in TCGA training set. **(A)** The distribution of survival time and risk score. **(B)** The 3 independent prognostic genes’ expression panel based on constructed risk score and clinical features. **(C)** The predictive value of this risk model for the 3-year (AUC, 0.676) and 5-year (AUC, 0.644) OS of patients with LUAD. **(D)** OS analysis for the two risk patterns based on 477 patients with LUAD. **(E)** RFS analysis for the two risk patterns based on training set. OS analysis for the two risk patterns in female **(F)** and male patients **(G)**.

Subsequently, we built a risk model according to a backwards stepwise Cox proportional hazards regression analysis: risk score = (-0.32738)*EXP_TMEM273_+ 0.25554*EXP_TMEM164_+(-0.29264)* EXP_TMEM125_. This formula was used to calculate the risk score of each patient. The distribution of survival time and risk score were shown in **Figure 2A**. **Figure 2B** shows the relationship between the expression characteristics of the finally identified 3 genes and risk score and clinical characteristics. In order to prove the performance of this model, the area under the ROC curve was calculated. The result showed that the AUC values for predicting 3-year and 5-year OS were 0.676 and 0.644, respectively (**Figure 2C**). The prognostic analysis for the two patterns (high-risk group and low-risk group divided by the median value of the risk score) based on 477 patients with LUAD in the TCGA set indicated a particularly prominent OS advantage in the low-risk group (**Figure 2D**) (HR: 0.5008, 95% confidence interval (CI): 0.3708–0.6764, P<0.0001). The Kaplan–Meier analysis for the two risk patterns (high-risk group and low-risk group divided by the optimal cutoff point of the risk score) based on the TCGA dataset also revealed a particularly prominent RFS advantage in the low risk-group (**Figure 2E**) (HR: 0.7216, 95% CI: 0.5265–0.9889, P=0.035). A lower risk score among female patients still had a better OS (**Figure 2F**) (HR: 0.5344, 95% CI: 0.3528–0.8067, P=0.0029). Similarly, male patients with a high-risk score had a significantly shorter survival than that with a low-risk score (**Figure 2G**) (HR: 2.151, 95% CI: 1.382–3.35, P=0.0007).

### Validation of the Predictive Value of This TMEM-Based Signature by Another Three Independent Cohorts

**Figure 3:**
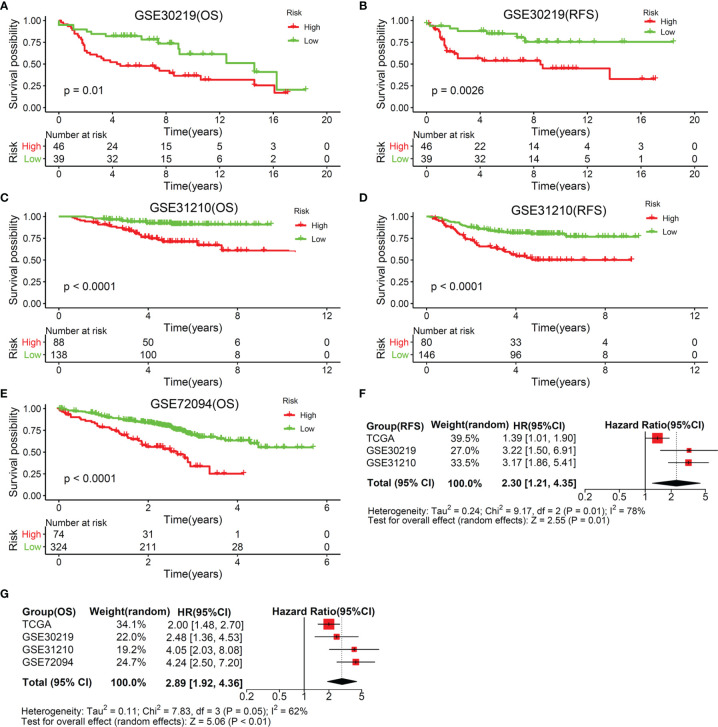
Validation of the predictive value of this TMEM-based signature by another three independent cohorts. OS **(A)** and RFS **(B)** analysis for the two risk patterns based on 85 LUAD patients collected from GSE30219 cohort. OS **(C)** and RFS **(D)** analysis for the two risk patterns based on 226 LUAD patients derived from GSE31210 cohort. **(E)** OS analysis for the two risk patterns based on 420 LUAD patients collected from GSE72094 cohort. **(F)** Meta‐analysis of RFS prognostic values of this TMEM-based signature for LUAD patients according to 3 cohorts (TCGA, GSE30219, and GSE31210). **(G)** Meta‐analysis of OS prognostic values of this TMEM-based signature for LUAD patients according to 4 cohorts (TCGA, GSE30219, GSE31210, and GSE72094).

To verify the stability and repeatability of this TMEM-based signature, risk scores of 731 patients with LUAD collected from three independent cohorts were calculated by the same formula. **Table S3** showed the basic information of those patients. According to the optimal risk score cut-off value, patients in the three cohorts were included in the high-risk group and the low-risk group, respectively. Compared with patients with a high- risk score, patients with a low-risk score showed a prominent OS benefit, either in GSE30219 (**Figure 3A**) (cut-off value: -3.5262, HR: 2.48, 95% CI: 1.36-4.53, P=0.01), GSE31210 (**Figure 3C**) (cut-off value: -910.7293, HR: 4.05, 95% CI: 2.03-8.08, P<0.001), or GSE72094 (**Figure 3E**) (cut-off value: -2.5451, HR: 4.24, 95% CI: 2.50-7.20, P<0.001). Next, a prognostic meta-analysis based on TCGA and GEO datasets (4 independent cohorts containing 1,208 cases) was performed, and the result verified that high score was a risk factor affecting the OS of patients with LUAD (**Figure 3G**) (HR: 2.89, 95% CI: 1.82–4.38, P<0.01). Similarly, we analyzed the impact of this risk score on RFS. High-risk patients showed worse RFS, either in GSE30219 (**Figure 3B**) (cut-off value: -3.5262, HR: 3.22, 95% CI: 1.50–6.91, P=0.003) or GSE31210 (**Figure 3D**) (cut-off value: -887.3541, HR: 3.17, 95% CI: 1.86–5.41, P<0.001). Moreover, a prognostic meta-analysis based on TCGA and GEO datasets (3 independent cohorts containing 788 cases) was also performed, and the result verified that the high score was a risk factor affecting the RFS of patients with LUAD (**Figure 3F**) (HR: 2.30, 95% CI: 1.21–4.35, P=0.01).

### The Performance of TMEM-Based Signature in Different Clinical Subgroups

In order to further verify the importance and repeatability of the model, we estimated the predictive power of this signature for the OS of patients with different disease stages, ages, N stages, T stages, and treatment methods. As expected, regardless of the subgroup, high-risk patients showed a significantly poorer OS (**Figure S2**).

### High Score of This TMEM-Based Signature Is an Independent Risk Factor

We have previously proven that this TMEM-based signature had a strong predictive power for the OS and RFS of patients with LUAD. Next, in order to further prove whether a high score was an independent risk factor for the poor prognosis of patients with LUAD, we performed univariate and multivariate Cox regression analyses. The result indicated that the age, TNM stage, STK11 mutation status, and risk score were proven to be independent prognostic predictors (**Table 1**).

**Table 1:** Univariate and multivariate Cox regression analysis for three-TMEM-based signature and clinical features in TCGA dataset.

	Univariable analysis			Multivariable analysis		
Characteristics	HR	95%CI	*P-*value	HR	95%CI	*P-*value
Age						
≤65 or >65	1.462	1.009–2.119	0.045	1.809	1.231–2.657	0.003
Gender						
Female or Male	0.642	0.444–0.929	0.019	0.77	0.526–1.127	0.179
Smoking index						
Yes or No	1.105	0.851–1.434	0.455			
TNM stage						
I, II, III, or V	1.51	1.27–1.796	0	1.569	1.309–1.881	0
EGFR status						
MUT or WT	1.595	0.909–2.797	0.103			
KRAS status						
MUT or WT	1.282	0.848–1.938	0.239			
STK11 status						
MUT or WT	1.621	1.052–2.499	0.029	2.119	1.341–3.348	0.001
TP53 status						
MUT or WT	1.194	0.825–1.728	0.347			
Risk score						
High or low	1.887	1.489–2.392	0	1.94	1.53–2.46	0

### Biological Pathways Closely Related to the TMEM-Based Signature

**Figure 4:**
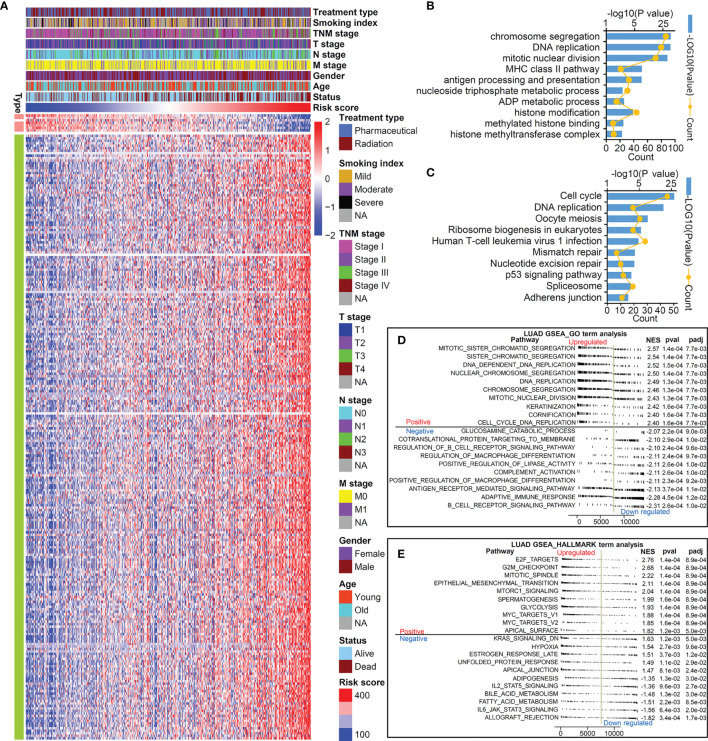
TMEM signature-related genes and pathways. **(A)** Heatmap of 251 genes significant correlated to risk score (|r|>4, P<0.05). KEGG **(B)** and GO **(C)** analysis of 1,030 genes correlated to risk score (|r|>0, P<0.05). GSEA GO term analysis **(D)** and hallmark term analysis **(E)** to compare the different enriched pathways between high-risk group and low-risk group.

Considering that this TMEM-based signature had excellent ability in predicting the prognosis of patients with LUAD, we further explore its potential mechanism. We first identified 251 genes closely correlated with the risk score using Spearman’s correlation analyses (Pearson |R|>0.4, *P*<0.05). As shown in **Figures 4A**, **7** genes were negatively correlated with the risk score and 244 genes were positively correlated with the risk score. In order to fully demonstrate the signaling pathways mediated by the genes correlated to this risk score, we performed KEGG (**Figure 4B**) and GO (**Figure 4C**) enrichment analyses on the 1,030 genes identified by the standard of Pearson |R|>0.3 and *P*<0.05. The result indicated that this TMEM-based signature was involved in cell proliferation, the immune signal pathway, metabolic process, and histone modification regulation.

Next, a GSEA analysis was performed to further compare the pathways involved in the high-risk group and low-risk group. The results demonstrated that biological pathways accelerating cell proliferation and promoting EMT were activated in the high-risk group, while immune pathways (such as adaptive immune response and complement activation) were blocked in the high-risk group (**Figures 4D, E**).

**Figure 5:**
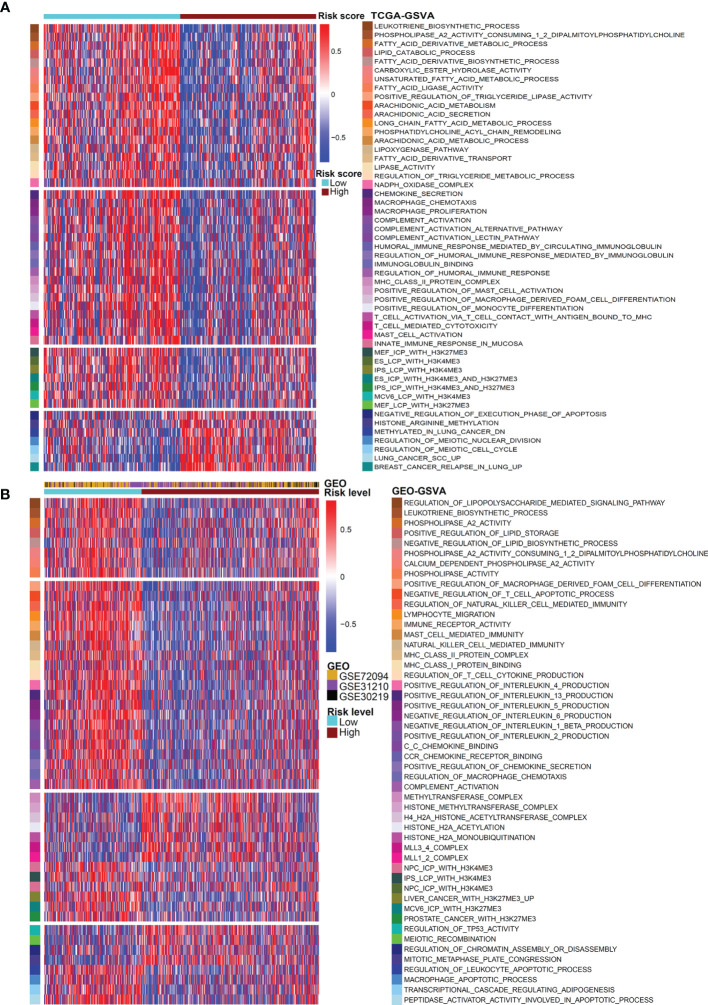
GSVA analysis for the two risk patterns. Heatmap visualizing these most important biological pathways in distinct risk patterns based on TCGA dataset **(A)** and GEO dataset **(B)**. Blue indicates repressed pathways, and red represents activated pathways.

Then, we performed GSVA analysis based on the gene expression matrix of LUAD patients in the TCGA database (**Figure 5A**). The low-risk group was prominently enriched in the biological pathways of fatty acid decomposition and synthesis of its derivatives, immune activation, and histone trimethylation modification, while the high-risk group was markedly related to cell proliferation, immunosuppression, low histone trimethylation modification, and so on. In addition, the potential mechanism found above was verified by 3 independent cohorts (GSE72094, GSE31210, and GSE30219) from the GEO database. We use the R packages of “sva” and “dplyr” to standardize and normalize these three expression matrices and then use the same algorithm for GSVA analysis. As shown in **Figure 5B**, compared with the high-risk group, the low-risk group was also enriched in the biological pathways of fatty acid decomposition and synthesis of its derivatives, immune activation, histone trimethylation modification, cell apoptosis, and cell proliferation inhibition.

### The Relationship Between This TMEM-Based Signature and Histone Trimethylation Modification in Patients With LUAD

**Figure 6:**
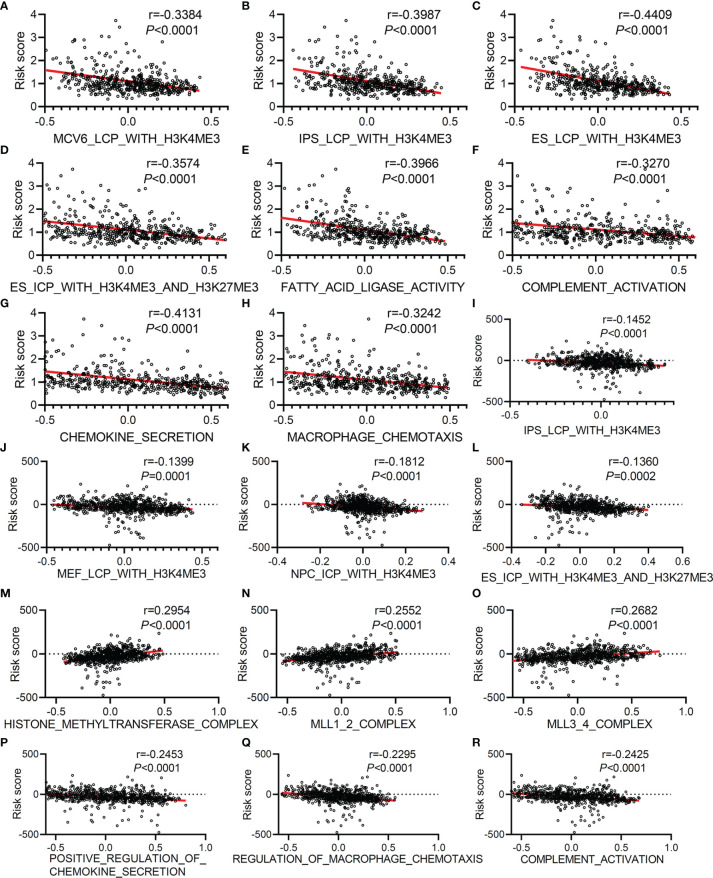
The correlation analysis between risk score and histone trimethylation modification, immune activity, and fatty acid metabolism based on GSVA analysis of gene expression data collected from TCGA and GEO. Correlation analysis between risk score and MCV6_LCP_WITH_H3K4ME3 **(A)**, IPS_LCP_WITH_H3K4ME3 **(B)**, ES_LCP_WITH_H3K4ME3 **(C)**, ES_ICP_WITH_H3K4ME3_AND_H3K27ME3 **(D)**, FATTY_ACID_LIGASE_ACTIVITY **(E)**, COMPLEMENT_ACTIVATION **(F)**, CHEMOKINE_SECRETION **(G)**, and MACROPHAGE_CHEMOTAXIS **(H)** based on GSVA analysis of gene expression data collected from TCGA. Correlation analysis between risk score and IPS_LCP_WITH_H3K4ME3 **(I)**, MEF_LCP_WITH_H3K4ME3 **(J)**, NPC_ICP_WITH_H3K4ME3 **(K)**, ES_ICP_WITH_H3K4ME3_AND_H3K27ME3 **(L)**, HISTONE_METHYLTRANSFERASE_COMPLEX **(M)**, MLL1_2_COMPLEX **(N)**, MLL3_4_COMPLEX **(O)**, POSITIVE_REGULATION_OF_CHEMOKINE_SECRETION **(P)**, REGULATION_OF_MACROPHAGE_CHEMOTAXIS **(Q)** and COMPLEMENT_ACTIVATION **(R)** based on GSVA analysis of normalized gene expression data collected from GEO (GSE30219, GSE31210, and GSE72094).

Since our first discovery of histone trimethylation modification and immune activation were the keys to the powerful performance of this TMEM-based signature predicting tumor prognosis, we further analyzed the correlation between this risk score and these two factors, as well as the effect of these factors on the prognosis of patients with LUAD. According to the GSVA analysis based on TCGA gene expression data, we found that the risk score was negatively correlated with MCV6_LCP_WITH_H3K4ME3 (**Figure 6A**), r=-0.3384, *P*<0.0001), IPS_LCP_WITH_H3K4ME3 (**Figure 6B**), r=-0.3987, *P*<0.0001), ES_LCP_WITH_H3K4ME3 (**Figure 6C**), r=-0.4409, *P*<0.0001), ES_ICP_WITH_H3K4ME3_and_H3K27ME3 (**Figure 6D**), r=-0.3574, *P*<0.0001), fatty acid ligase activity (**Figure 6E**), r=-0.3966, *P*<0.0001), complement activation (**Figure 6F**), r=-0.327, *P*<0.0001), chemokine secretion (**Figure 6G**), r=-0.4131, *P*<0.0001), and macrophage chemotaxis (**Figure 6H**), r=-0.3242, *P*<0.0001). Similarly in GEO datasets, the risk score was negatively correlated with IPS_LCP_WITH_H3K4ME3(**Figure 6I**), r=-0.1452, *P*<0.0001), MEF_LCP_WITH _H3K4ME3(**Figure 6J**), r=-0.1399, *P*<0.0001), NPC_ICP_ WITH_H3K4ME3(**Figure 6K**), r=-0.1812, *P*<0.0001), ES_ICP_ WITH_H3K4ME3_AND_H3K27ME3(**Figure 6L**), r=-0.136, *P*<0.0001), the positive regulation of chemokine secretion (**Figure 6P**), r=-0.2453, *P*<0.0001), regulation of macrophage chemotaxis (**Figure 6Q**), r=-0.2295, *P*<0.0001), and complement activation (**Figure 6R**), r=-0.2425, *P*<0.0001) and was positively correlated with HISTONE_METHYLTRANSFERASE_ COMPLEX (**Figure 6M**), r=0.2954, *P*<0.0001), MLL1_2_COMPLEX (**Figure 6N**), r=0.2552, *P*<0.0001), and MLL3_4_COMPLEX(**Figure 6O**), r=0.2682, *P*<0.0001).

Next, we evaluated the effects of histone trimethylation modification and immune activation on patients’ OS. Based on TCGA data analysis, we found that LUAD patients with high levels of H3K4me3 presented a particularly prominent survival advantage (**Figures S3A, B**), and high levels of macrophage chemotaxis (**Figure S3C**) and T-cell- mediated cytotoxicity (**Figure S3D**) were also beneficial to OS. In GEO data, among 731 cases, patients with high level of methyltransferase (MLL1_2_COMPLEX and HISTONE_METHYLTRANSFERASE_COMPLEX) had poorer OS (**Figures S3E,F**). Similar to TCGA results, the patients collected from GEO with a high level of POSITIVE_ REGULATION_OF_CHEMOKINE_SECRETION (**Figure S3G**) and MHC_CLASS_II_PROTEIN_COMPLEX (**Figure S3H**) presented a particularly prominent survival advantage. According to the above research results, we found that the histone hypotrimethylation level, decreased secretion of chemokines, macrophage chemotaxis inhibition, and immunosuppression were closely related to the poor prognosis of patients with LUAD, and we speculated that the reason that a TMEM-based signature is used to predict the prognosis of patients with LUAD was due to abnormal histone trimethylation modifications, immunosuppression, and macrophage chemotaxis inhibition mediated by TMEM125, TMEM164, and TMEM273.

### Immune Cell Infiltration Characteristics in Distinct TMEM-Based Patterns

**Figure 7:**
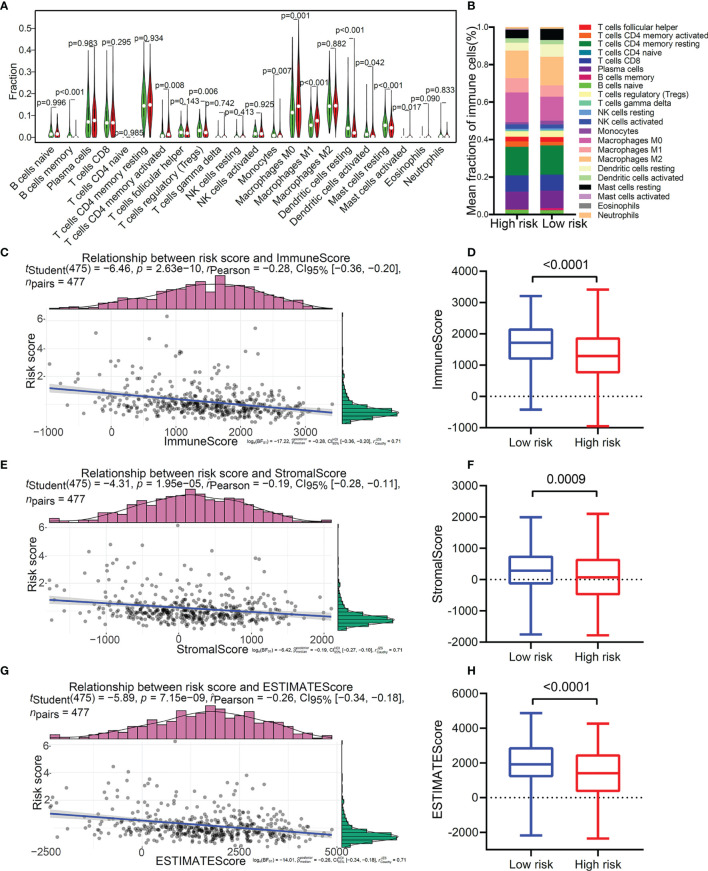
Immune cell infiltration profiles in the two distinct risk patterns. **(A)** The comparison of the abundance of each infiltrating immune cell between high-risk group and low-risk group. **(B)** The distribution of each infiltrating immune cell in the two risk patterns. **(C)** The correlation analysis of risk score and immune score. **(D)** The comparison of immune score between high-risk group and low-risk group. **(E)** The correlation analysis of risk score and stromal score. **(F)** The comparison of stromal score between the two risk patterns. **(G)** The correlation analysis of risk score and ESTIMATE score. **(H)** The comparison of ESTIMATE score between the two risk patterns.

As we all know, the proportion and distribution of immune cells in the TME were related to immune surveillance and immune function. In view of this, we analyzed the immune cell infiltration in the TME of patients with LUAD. The result suggested that patients with a high score were enriched in T cells CD4 memory activated, macrophages M0, macrophages M1, and mast cells activated (**Figure 7A**), and patients with low score were remarkably rich in B cells memory, T cells regulatory (Tregs), monocytes, dendritic cells resting, dendritic cells activated, and mast cells resting (**Figure 7B**). The distribution characteristics of immune cells is shown in **Figure 7B**). In addition, correlation analysis further confirmed that patients with a different risk score showed specific characteristics of immune cell infiltration (**Figures S4A-J**). We further used the ESTIMATE algorithm to evaluate the TME immune status of high-risk and low-risk patients. The results showed that this TMEM-based risk score was negatively correlated with immunescore (**Figure 7C**), stromalscore (**Figure 7E**), and ESTIMATEScore (**Figure 7G**). Compared with the low-risk group, the high-risk group had lower immunescore (**Figure 7D**), stromalscore (**Figure 7F**), and ESTIMATEScore (**Figure 7H**). Furthermore, we found that patients with high immunescore (**Figure S4K**) or ESTIMATEScore (**Figure S4L**) presented a particularly prominent survival advantage. These results indicated from a deeper level that the poor prognosis of patients in the high-risk group may be related to the inactivation of immune cell function in the TME.

### Comparison of the Prognostic Ability of This TMEM-Based Signature and TIDE Score on Patients With LUAD

Immunotherapy is already the first-line treatment for LUAD. To evaluate whether this TMEM-based signature could be used to predict tumor immunotherapy response, we compared this risk signature with the currently recognized biomarkers. As shown in **Figure S5A**, patients with a high-risk score had a significant mutation load. It has been widely proven that patients with high TMB were more sensitive to immunotherapy (35). Our result also proved that the risk score was positively correlated with TMB (**Figure S5B**), which meant that patients with a high-risk score were more inclined to benefit from immunotherapy because they had a higher mutation load. TIDE is the newly discovered biomarker with a tumor prognosis prediction ability and tumor immunotherapy response prediction ability (32). Therefore, we compared the predictive power of this TMEM-based signature with TIDE, IFNG, the dysfunction score, exclusion score, and CD8 score in the OS of patients with LUAD. As a result, the predictive value of this risk signature was better than other biomarkers, no matter for 1-year OS (**Figures S6A, B**), 3-year OS (**Figures S6C, D**), or 5-year OS (**Figures S6E, F**) of patients with LUAD.

### Relationship Between the Distinct TMEM-Based Patterns and Immunotherapy Response on LUAD Patients With Different Mutation Type

ICI therapy is currently the most important tumor immunotherapy method. In order to further study that this signature predicted the effect of ICI therapy in LUAD patients with different common mutation types, we did a correlation analysis between the risk score of genetic mutation and non-mutation patients and the expression of immune checkpoints. The results indicated that the risk score of LUAD patients was positively correlated with the expression of CD276 (**Figures S7A-H**). The risk score was negatively correlated with the expression of HAVCR2 and ICOS among LUAD patients with EGFR wild type (**Figure S7B**), KRAS wild type (**Figure S7D**), STK11 wild type (**Figure S7F**), or TP53 mutation (**Figure S7G**).

## Discussion

Lung cancer is still one of the malignant tumors with the highest morbidity and mortality (36, 37). Although the current treatment of lung cancer has achieved amazing results, the 5-year survival rate of lung cancer is still far from expected. Current treatment strategies can only be effective for a small number of patients, and no breakthrough has been made in precision treatment strategies (38, 39). In this study, for the first time, we comprehensively analyzed the mechanism of TMEM molecular family in LUAD and described the immune characteristics of the TME based on the expression level of TMEMs. Meanwhile, we constructed a risk model for predicting the prognosis of LUAD based on the expression of these three identified TMEMs, which demonstrated a strong predictive ability as it withstood the validation of multiple independent cohorts. In addition, based on the GSVA analysis of the transcriptomics data of 1,208 LUAD patients from the TCGA and GEO databases, we innovatively found the characteristics of histone trimethylation modification in the TME of LUAD patients. The level of H3K4me3 is significantly negatively correlated with the risk scores we identified, and compared with LUAD patients with a low level of H3K4me3, patients with a high level of H3K4me3 showed a significant OS advantage. More importantly, through comparative analysis with the current immunotherapy response biomarkers, we demonstrated for the first time the most suitable ICI treatment options for LUAD patients with different mutation types.

Although the members of the molecular family of TMEMs are very large, there are not many research reports on TMEMs in the field of tumors or tumor immunotherapy. Through a literature search, we only found a few reports of TMEMs in tumor immunotherapy (40–42). The most widely reported is TMEM173, known as STING, which participates in tumor immunity by regulating the natural immune response mediated by the cGAS-STING signaling pathway (43). Based on whole-genome sequencing data, using multiple cohorts and large samples, this study comprehensively analyzed the role and mechanism of TMEMs in LUAD for the first time. The research results pointed out the direction and laid the foundation for the subsequent research on TMEMs.

This TMEM-based signature was generated using Cox regression analysis and LASSO regression analysis, and its performance was validated by four independent cohorts and clinical subgroups. In order to explore the underlying molecular mechanism of the poor prognosis of patients in the high-risk group, we performed GO and KEGG analyses on the genes that have a significant correlation with the risk score and performed GSEA and GSVA analyses on the sequencing data of patients in the high-risk group and low-risk group. Biological pathway analysis indicated that the biological process of anti-tumor immune response (innate immune response, T-cell activation, T- cell-mediated cytotoxicity, macrophage chemotaxis, antigen processing and presentation, etc.) in the high-risk group was inhibited, while the pathways that promoted cell proliferation (cell cycle, DNA replication, mitotic nuclear division, chromosome segregation, meiotic nuclear division, etc.) were significantly activated. We also found the differences in fatty acid metabolism between high-risk patients and low-risk patients. The high-risk group showed a significant reduction in lipase activity and fatty acid catabolism disorders. TME ESTIMATEScore analysis further proved that patients with a high-risk score were in an immunosuppressive state with a low level of immune score and stromal score. All these findings fully explained the reasons for the poor prognosis of patients in the high-risk group.

H3K4me3 is one of the most recognized epigenetic modifications that regulate gene transcription. The enrichment of H3K4me3 in the promoter region of oncogenes is an important factor of tumor occurrence and progression (44–46). Kim and his colleagues found that the low expression of MLL2 inhibited the proliferation of lung cancer cells by downregulating H3K4me3 (47). In addition, studies have shown that the cooperation of KDM6A and KMT2B promoted tumorigenesis by increasing the expression of H3K4me3 (48). All the above studies have shown that H3K4me3 played a role in promoting cancer. However, H3K4me3 demethylation caused by the loss of KMT2D function in the germinal center of lymphoma led to rapid tumor progression, and the re-establishing of H3K4 trimethylation caused significant tumor growth inhibition (49). It was also reported that KDM5B downregulated PTEN expression by suppressing the accumulation of H3K4me3 in the PTEN promoter region so as to enhance the radioresistance of NSCLC (50). Therefore, the current molecular biology experiments have shown the complex and contradictory functional characteristics of H3K4me3 in tumors. In our study, we discovered for the first time the H3K4me3 modification characteristics of 1,208 patients with LUAD. The risk score was negatively correlated with the H3K4me3 level, which means that LUAD patients with a high-risk score tended to have a lower H3K4me3 level. Interestingly, this result was consistent with the OS calculated based on the H3K4me3 level among TCGA cases. Unfortunately, although in the validation set based on three independent GEO cohorts, the correlation between the risk score and the H3K4me3 level was consistent with that of the training set, patients with high H3K4me3 levels did not show a clear survival advantage in the validation set. Our multi-cohort and large sample study results indicated that H3K4me3 modification had the specific effect of regulating LUAD progression. Although this study was only based on sequencing data from public databases, it was the largest sample of studies on the relationship between the epigenetic modifications of H3K4me3 and tumor prognosis.

Another important finding was the potential key role of this TMEM-based signature in guiding the treatment of ICIs in patients with a different mutation type of LUAD. We found that CD276 had a strong positive correlation with the risk score, regardless of whether there were mutations in these designated genes. It suggested that high risk-score patients may be suitable for treatment with anti-CD276. Similarly, the risk score had a strong negative correlation with HAVCR2 and ICOS among patients with EGFR-WT, KRAS-WT, STK11-WT, or TP53-MUT, and we speculated that patients with these mutation types with low scores were suitable for treatment with HAVCR2 or ICOS inhibitors. These innovative discoveries will help clinicians to implement more precise and targeted immunotherapy for patients with LUAD. In view of the fact that the study is mainly based on public databases, and the conclusions are indirect, prospective clinical research is needed for further verification.

Although this research has innovative clinical guidance value, there are still some shortcomings that need to be clarified. First of all, KRAS and EGFR mutations have profound effects on the treatment and prognosis of lung cancer patients. In this study, we did not find a cohort of lung cancer patients with KRAS and EGFR mutations, so it is impossible to study whether KRAS and EGFR mutations will bias the results. Second, this signature is constructed on the basis of sequencing and microarray data, and there is a lack of verification of fresh-frozen tissues. Third, we use GSVA analysis to obtain the H3K4me3 level of each sample, and direct protein quantification is required to determine the H3K4me3 expression level. Fourth, the therapeutic effect of this TMEM-based signature guiding the treatment of ICIs in LUAD patients with different gene mutation status needs to be verified with a prospective cohort.

In conclusion, for the first time, we have made a comprehensive analysis of the expression characteristics and mechanism of TMEMs in LUAD and constructed a risk model based on the expression of identified TMEMs to predict the prognosis of LUAD. Based on a large clinical sample and multiple cohorts, we innovatively discovered the H3K4me3 modification characteristics of patients with LUAD. These newly discovered immunological and epigenetic features provide us with a solid foundation for an in-depth understanding of the TME of LUAD and provide strategies to better achieve a precise treatment of LUAD and improve the therapeutic effect of ICIs.

## Data Availability Statement

The datasets presented in this study can be found in online repositories. The names of the repository/repositories and accession number(s) can be found in the article/**Supplementary Material**.

## Author Contributions

TF, YL, HL, YZ, and LW designed the study, performed data collection, and conducted data analysis. TF, HT, BZ, and LX drafted the manuscript. TF, CL and JH revised this paper. CL and JH supervised and funded this study. All authors contributed to the article and approved the submitted version.

## Funding

This work was supported by the National Natural Science Foundation of China (81972196), the CAMS Innovation Fund for Medical Sciences (CIFMS) (2021-1-I2M-012), the National Key R&D Program of China (2020AAA0109505, YS2021YFF120009), and the R&D Program of Beijing Municipal Education commission (KJZD20191002302).

## Conflict of Interest

The authors declare that the research was conducted in the absence of any commercial or financial relationships that could be construed as a potential conflict of interest.

## Publisher’s Note

All claims expressed in this article are solely those of the authors and do not necessarily represent those of their affiliated organizations, or those of the publisher, the editors and the reviewers. Any product that may be evaluated in this article, or claim that may be made by its manufacturer, is not guaranteed or endorsed by the publisher.
