# Supplementary material for: Transmembrane Protein-Based Risk Model and H3K4me3 Modification Characteristics in Lung Adenocarcinoma
Source: Front Oncol. 2022 Mar 22;12:828814. doi: 10.3389/fonc.2022.828814 (PMC8980838; doi:10.3389/fonc.2022.828814)
Supplement: Supplementary file 1 [file DataSheet_1.docx]

| **Table S1. Differentially expressed genes (DEGs) between normal and tumor tissues** | | | | |
| --- | --- | --- | --- | --- |
| Genes | logFC | logCPM | *P* Value | FDR |
| TMEM100 | -4.312145239 | 12.17179468 | 2.05E-149 | 5.11E-147 |
| TMEM204 | -1.961772317 | 12.41779597 | 1.54E-102 | 1.91E-100 |
| TMEM88 | -2.142198602 | 11.13417765 | 1.34E-75 | 1.11E-73 |
| TMEM47 | -1.761497246 | 11.75544974 | 3.29E-65 | 2.05E-63 |
| TMEM273 | -1.552105802 | 11.18858231 | 1.65E-55 | 8.20E-54 |
| TMEM150B | -1.811914139 | 10.88907322 | 4.68E-47 | 1.94E-45 |
| TMEM184A | 2.89248592 | 11.68802527 | 7.32E-40 | 2.60E-38 |
| TMEM139 | -1.91387381 | 11.07485968 | 2.46E-29 | 6.82E-28 |
| TMEM177 | 1.498175233 | 11.42016553 | 6.63E-29 | 1.65E-27 |
| TMEM140 | -0.843841311 | 12.5875193 | 2.49E-27 | 5.63E-26 |
| TMEM164 | -0.973838124 | 12.42645988 | 2.66E-26 | 5.52E-25 |
| TMEM220 | -1.298444799 | 10.61751591 | 2.67E-25 | 5.11E-24 |
| TMEM45B | 1.803194128 | 13.16392319 | 2.33E-24 | 4.14E-23 |
| TMEM132A | 1.948813616 | 12.08740659 | 8.05E-24 | 1.34E-22 |
| TMEM74B | -1.418045119 | 10.49874518 | 2.55E-22 | 3.96E-21 |
| TMEM62 | 1.013388811 | 12.31847178 | 1.09E-21 | 1.59E-20 |
| TMEM69 | 0.836406723 | 12.47760797 | 2.75E-20 | 3.80E-19 |
| TMEM63C | 3.1776184 | 10.72369167 | 3.44E-19 | 4.51E-18 |
| TMEM190 | -2.20639243 | 11.32876726 | 5.88E-17 | 6.97E-16 |
| TMEM79 | 1.237811701 | 11.23489723 | 1.97E-16 | 2.23E-15 |
| TMEM59L | 3.894015566 | 12.22222488 | 1.74E-15 | 1.88E-14 |
| TMEM106C | 1.000801224 | 13.40851831 | 6.51E-15 | 6.24E-14 |
| TMEM156 | 2.203116737 | 11.00788491 | 1.68E-14 | 1.50E-13 |
| TMEM212 | -1.823168346 | 9.989281074 | 4.56E-14 | 3.91E-13 |
| TMEM243 | -0.814557222 | 12.41137318 | 1.65E-13 | 1.33E-12 |
| TMEM159 | 0.918335999 | 13.51362731 | 3.57E-13 | 2.69E-12 |
| TMEM125 | -0.875667837 | 14.3660429 | 4.81E-13 | 3.52E-12 |
| TMEM106B | 0.900255849 | 12.19632209 | 9.36E-13 | 6.66E-12 |
| TMEM92 | 1.215481167 | 11.87096057 | 2.07E-12 | 1.40E-11 |
| TMEM182 | 1.392372265 | 10.66576303 | 2.08E-12 | 1.40E-11 |
| TMEM52 | 1.72326656 | 10.6022852 | 4.49E-12 | 2.79E-11 |
| TMEM231 | -0.988863888 | 10.85192701 | 6.33E-12 | 3.76E-11 |
| TMEM206 | 0.833216972 | 11.4463061 | 5.43E-11 | 2.88E-10 |
| TMEM99 | 0.886316227 | 11.82581428 | 5.92E-11 | 3.07E-10 |
| TMEM171 | 2.162992122 | 10.35296814 | 1.78E-10 | 9.06E-10 |
| TMEM105 | 1.699772527 | 10.30269619 | 6.68E-10 | 3.20E-09 |
| TMEM132C | -1.522265097 | 9.841997645 | 7.02E-10 | 3.30E-09 |
| TMEM255B | -0.976137387 | 10.39033731 | 9.47E-10 | 4.37E-09 |
| TMEM255A | -1.00435031 | 10.35020034 | 2.18E-09 | 9.52E-09 |
| TMEM178A | -1.150869839 | 10.5768337 | 1.61E-08 | 6.36E-08 |
| TMEM232 | -1.329476737 | 9.911344479 | 2.56E-08 | 9.97E-08 |
| TMEM158 | 1.299882414 | 10.84896678 | 2.33E-07 | 8.27E-07 |
| TMEM61 | 1.164017537 | 10.93825173 | 2.52E-07 | 8.72E-07 |
| TMEM246 | -1.038109912 | 10.68962376 | 2.52E-07 | 8.72E-07 |
| TMEM176B | 0.833924995 | 14.84090648 | 5.42E-07 | 1.75E-06 |
| TMEM88B | 1.715631721 | 10.03861876 | 9.40E-07 | 2.93E-06 |
| TMEM176A | 0.893742466 | 13.35596959 | 1.41E-06 | 4.24E-06 |
| TMEM229A | 2.769083313 | 10.27418786 | 2.03E-06 | 6.03E-06 |
| TMEM145 | 1.720389149 | 10.05386 | 2.36E-06 | 6.92E-06 |
| TMEM236 | -0.948352926 | 10.01166614 | 2.68E-05 | 7.26E-05 |
| TMEM252 | -1.070587599 | 9.851530758 | 2.83E-05 | 7.57E-05 |
| TMEM117 | 0.842457735 | 10.49979694 | 4.12E-05 | 0.000108012 |
| TMEM82 | 1.588570458 | 9.918208035 | 6.04E-05 | 0.000153551 |
| TMEM45A | 0.934045753 | 11.69021243 | 8.16E-05 | 0.00019932 |
| TMEM198 | 0.807775272 | 10.55425869 | 0.000224838 | 0.000528158 |
| TMEM40 | 0.992083313 | 10.12154427 | 0.000896504 | 0.002011077 |
| TMEM270 | 0.880031301 | 10.04669553 | 0.002524436 | 0.005418834 |
| TMEM238L | 1.570754146 | 9.981277606 | 0.002736696 | 0.005678643 |
| TMEM179 | 1.238619863 | 9.872781231 | 0.004366185 | 0.008838863 |
| TMEM74 | 0.986278652 | 9.875062151 | 0.015660082 | 0.029766111 |
| TMEM178B | 0.818286681 | 10.03769814 | 0.021001418 | 0.038735948 |

| **Table S2. TMEMs related to the prognosis of LUAD screened by univariate Cox regression analysis** | | |
| --- | --- | --- |
| Genes | HR | *P* value |
| TMEM171 | 1.477404843 | 1.94E-05 |
| TMEM125 | 0.752700525 | 3.26E-05 |
| TMEM243 | 0.758495324 | 0.009943833 |
| TMEM63C | 0.832873469 | 0.018701135 |
| TMEM273 | 0.769145781 | 0.019501612 |
| TMEM158 | 1.195432217 | 0.019515182 |
| TMEM45A | 1.14408387 | 0.019645649 |
| TMEM231 | 0.74688931 | 0.025748906 |
| TMEM252 | 0.423695737 | 0.038199448 |
| TMEM164 | 1.270948646 | 0.038630967 |
| TMEM212 | 0.656734941 | 0.043158765 |

| **Table S3. Clinical characteristics of LUAD from multiple cohorts** | | | | |
| --- | --- | --- | --- | --- |
| Characteristics | TCGA cohort N=477 | GSE31210 N=226 | GSE72094 N=420 | GSE30219 N=85 |
| Age, year | 68(62-73) | 61(55-65) | 70(64-76) | 60(55-69) |
| Gender |  |  |  |  |
| male | 214 | 105 | 188 | 66 |
| female | 254 | 121 | 232 | 19 |
| Smoking |  |  |  |  |
| Yes | 390 | 111 | 320 | / |
| No | 66 | 115 | 31 | / |
| NA | 12 | 0 | 69 | / |
| Stage |  |  |  |  |
| I and II | 360 | 226 | 334 | 84 |
| III and IV | 100 | 0 | 80 | 1 |
| NA | 8 | 0 | 6 | 0 |
| Status |  |  |  |  |
| Alive | 291 | 191 | 298 | 40 |
| Death | 177 | 35 | 122 | 45 |
| EGFR status |  |  |  |  |
| MUT | 65 | 127 | 43 | - |
| WT | 412 | 99 | 377 | - |
| KRAS status |  |  |  |  |
| MUT | 114 | 20 | 144 | - |
| WT | 363 | 206 | 276 | - |
| STK11 status |  |  |  |  |
| MUT | 65 | - | 65 | - |
| WT | 412 | - | 355 | - |
| TP53 status |  |  |  |  |
| MUT | 233 | - | 103 | - |
| WT | 244 | - | 317 | - |


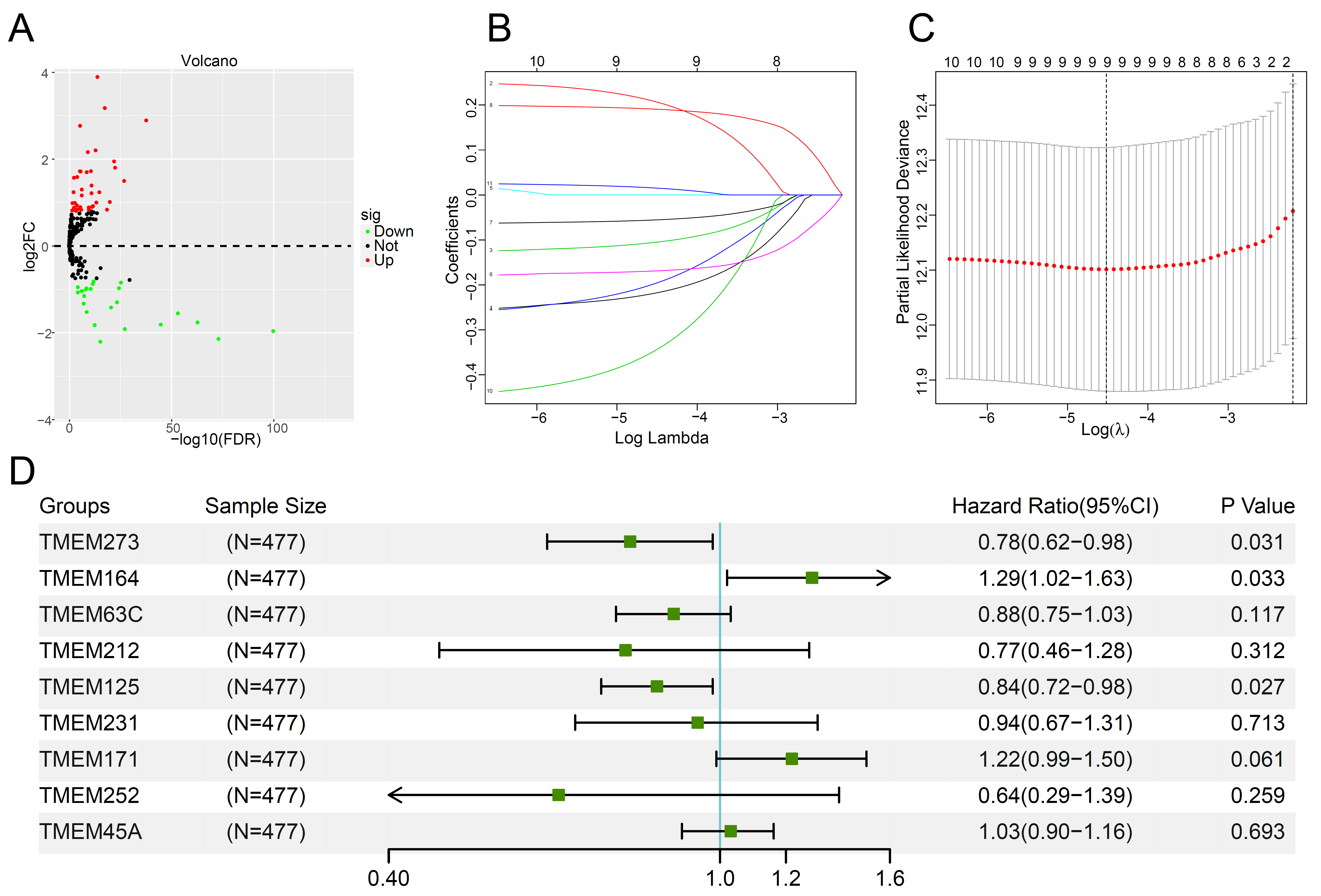


**Figure S1** Construction of TMEM-based signature for LUAD patients. **(A)** Differentially expressed TMEMs between normal and tumor tissues. **(B)** Coefficient analysis of the important TMEMs identified by univariate Cox analysis. **(C)** 100-fold cross-validation for selected parameters. **(D)** Multivariate Cox analysis for the identified 9 TMEMs.


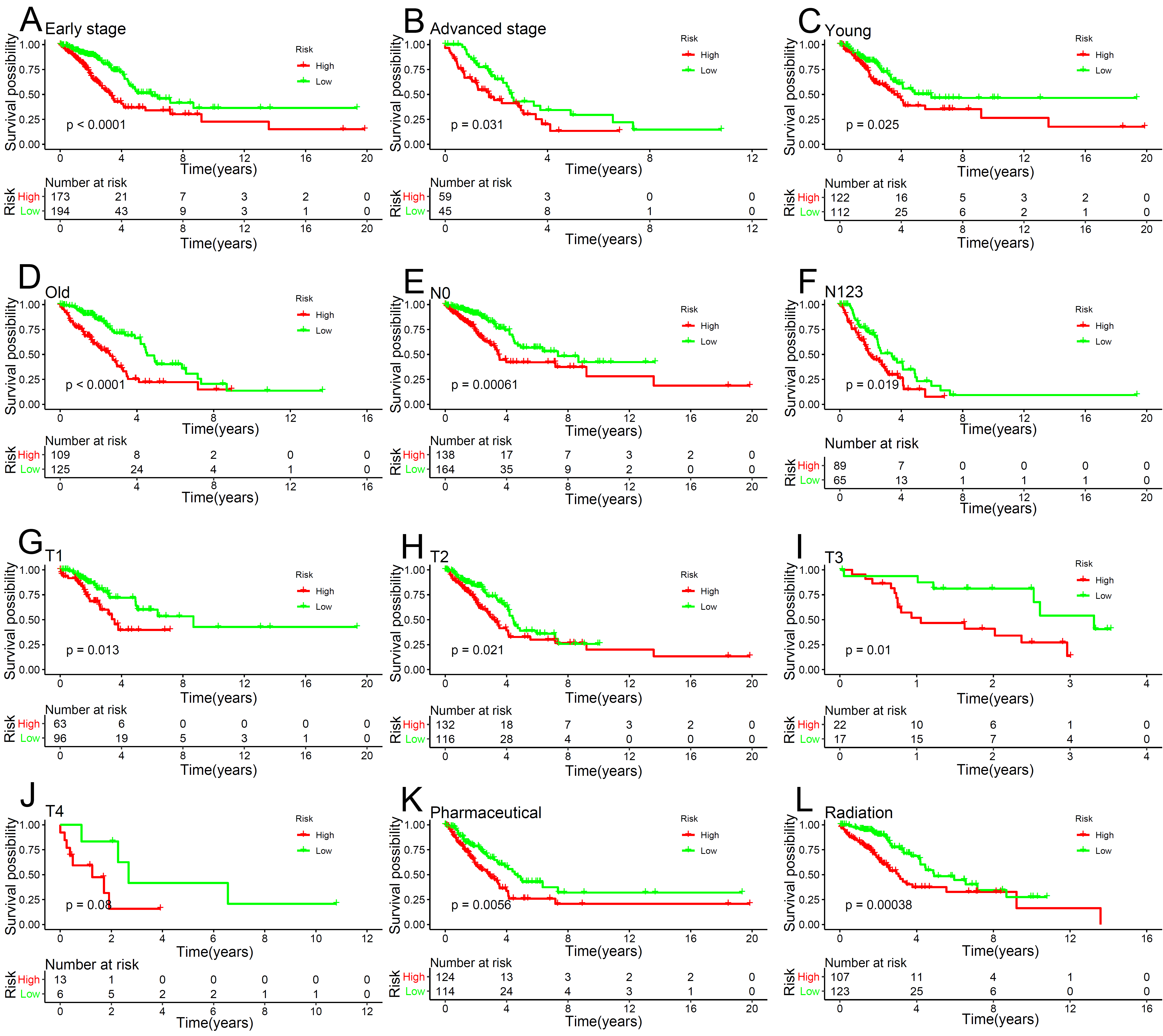


**Figure S2** The predictive value of this TMEM-based signature in LUAD patients with different clinical subtype. OS analysis for the two risk patterns based on LUAD patients with early stage **(A)**, advanced stage **(B)**, young **(C)**, old **(D)**, N0 stage **(E)**, N1/2/3 stage **(F)**, T1 stage **(G)**, T2 stage **(H)**, T3 stage **(I)**, T4 stage **(J)**, pharmaceutical-treated **(K)**, or radiation-treated **(L)**.


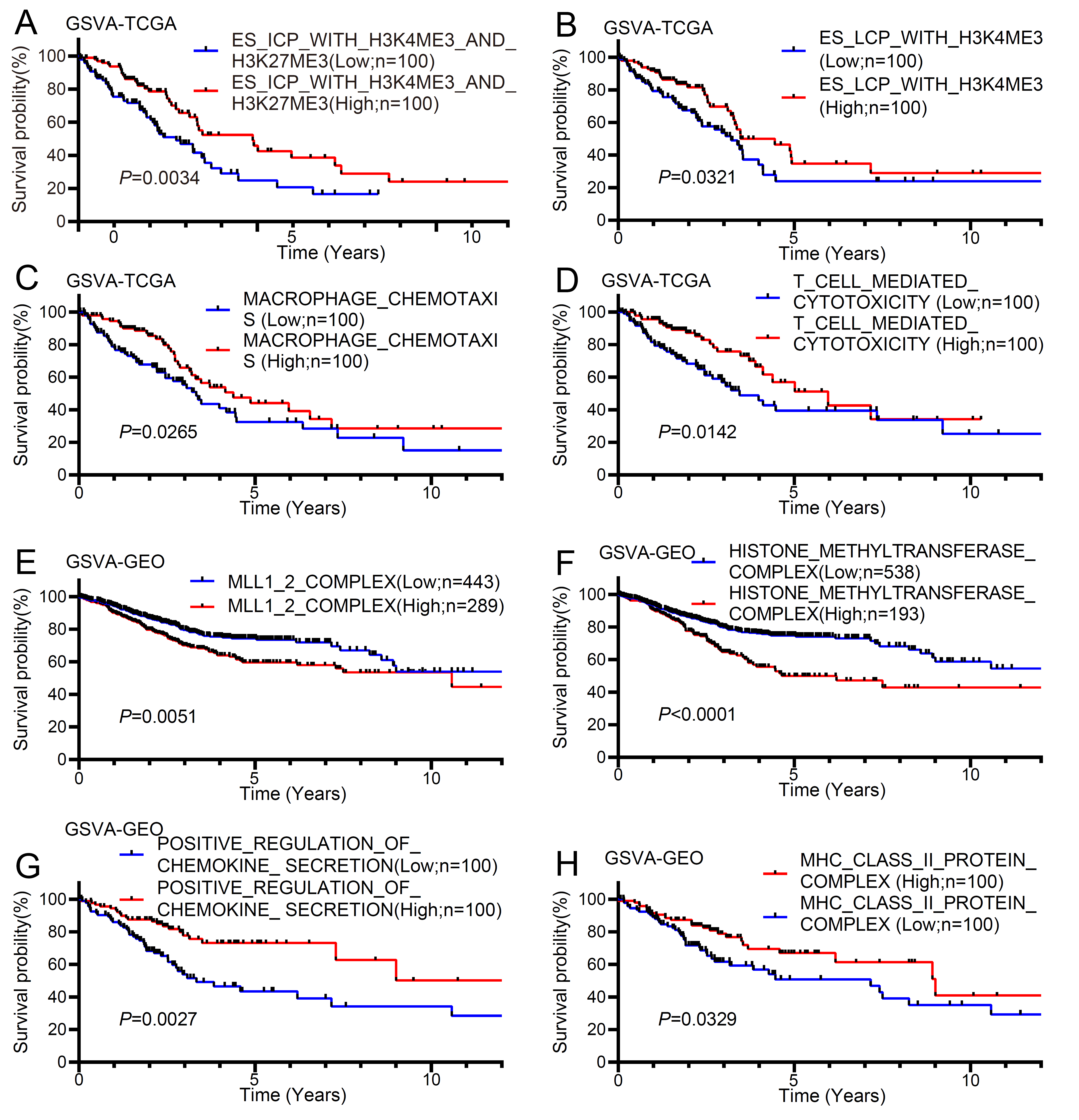


**Figure S3** The effect of histone modification and immune status on the prognosis of patients with LUAD. OS analysis for the high and low level of ES_ICP_WITH_H3K4ME3_AND_H3K27ME3 **(A)**, ES_LCP_WITH_H3K4ME3 **(B)**, MACROPHAGE_CHEMOTAXIS (**C**), and T_CELL_MEDIATED_CYTOTOXICITY (**D**) based on 477 patients in TCGA cohort. OS analysis for the high and low level of MLL1_2_COMPLEX (**E**), and MLL3_4_COMPLEX (**F**) based on 731 LUAD patients in three GEO cohorts. OS analysis for the high and low level of POSITIVE_REGULATION_OF_CHEMOKINE_SECRETION (**G**) and MHC_CLASS_II_PROTEIN_COMPLEX based on the three GEO cohorts (**H**).


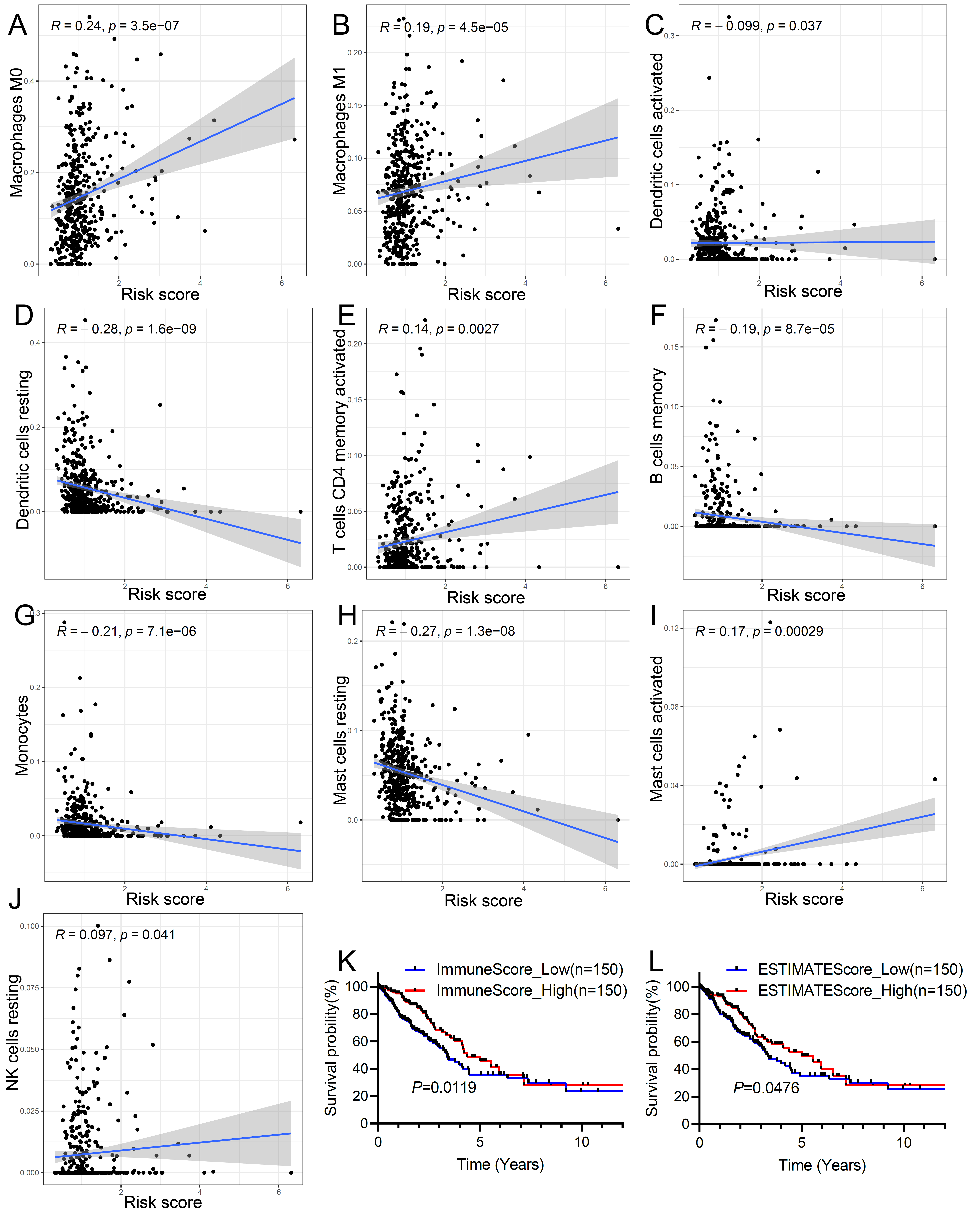


**Figure S4** The characteristics of immune cell infiltration in TME and its correlation with risk score. Correlation analysis between risk score and Macrophages M0 (**A**), Macrophages M1 (**B**), Dendritic cells activated (**C**), Dendritic cells resting (**D**), T cells CD4 memory activated (**E**), B cells memory (**F**), Monocytes (**G**), Mast cells resting (**H**), Mast cells activated (**I**), and NK cells resting (**J**). OS analysis for LUAD patients with different immune score (**K**) and ESTIMATE score (**L**).


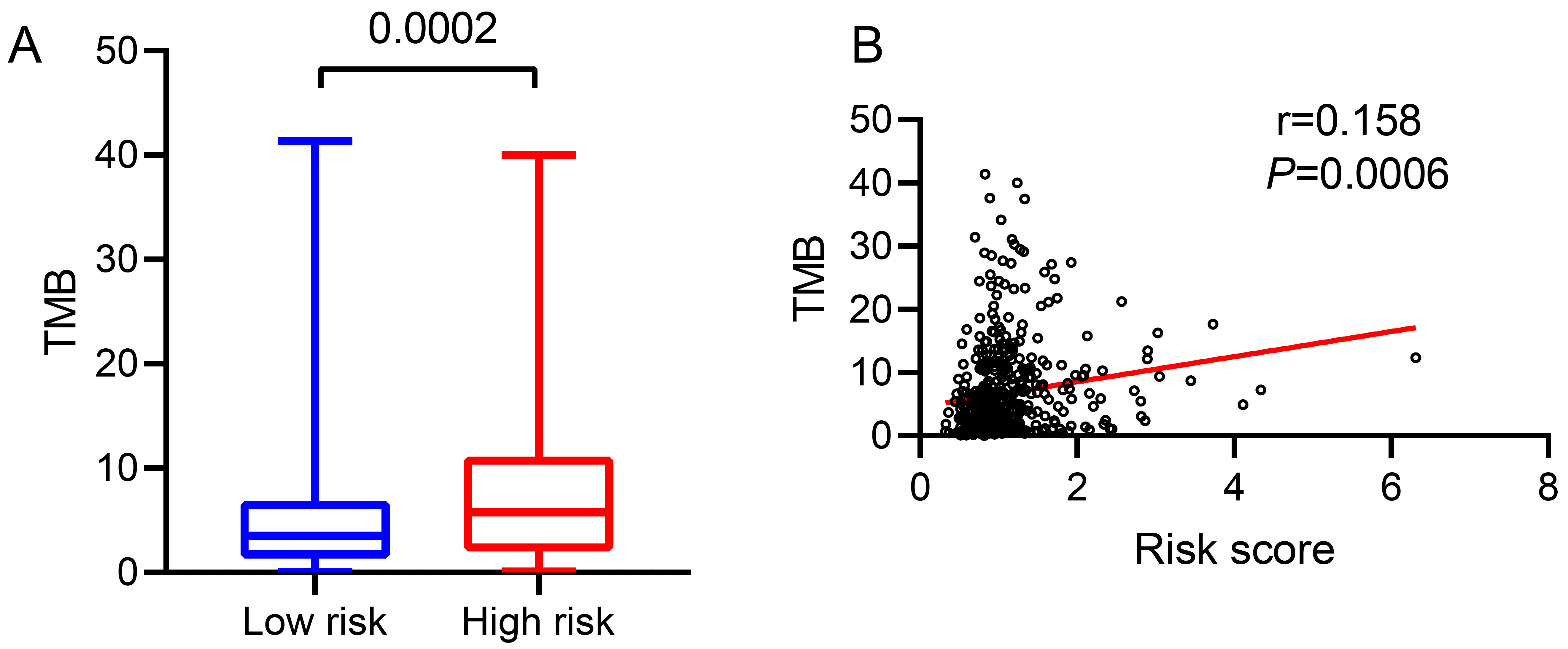


**Figure S5** The impact of TMB on immune score. **(A)** The comparison of TMB between high risk group and low risk group. **(B)** The correlation analysis between risk score and TMB.


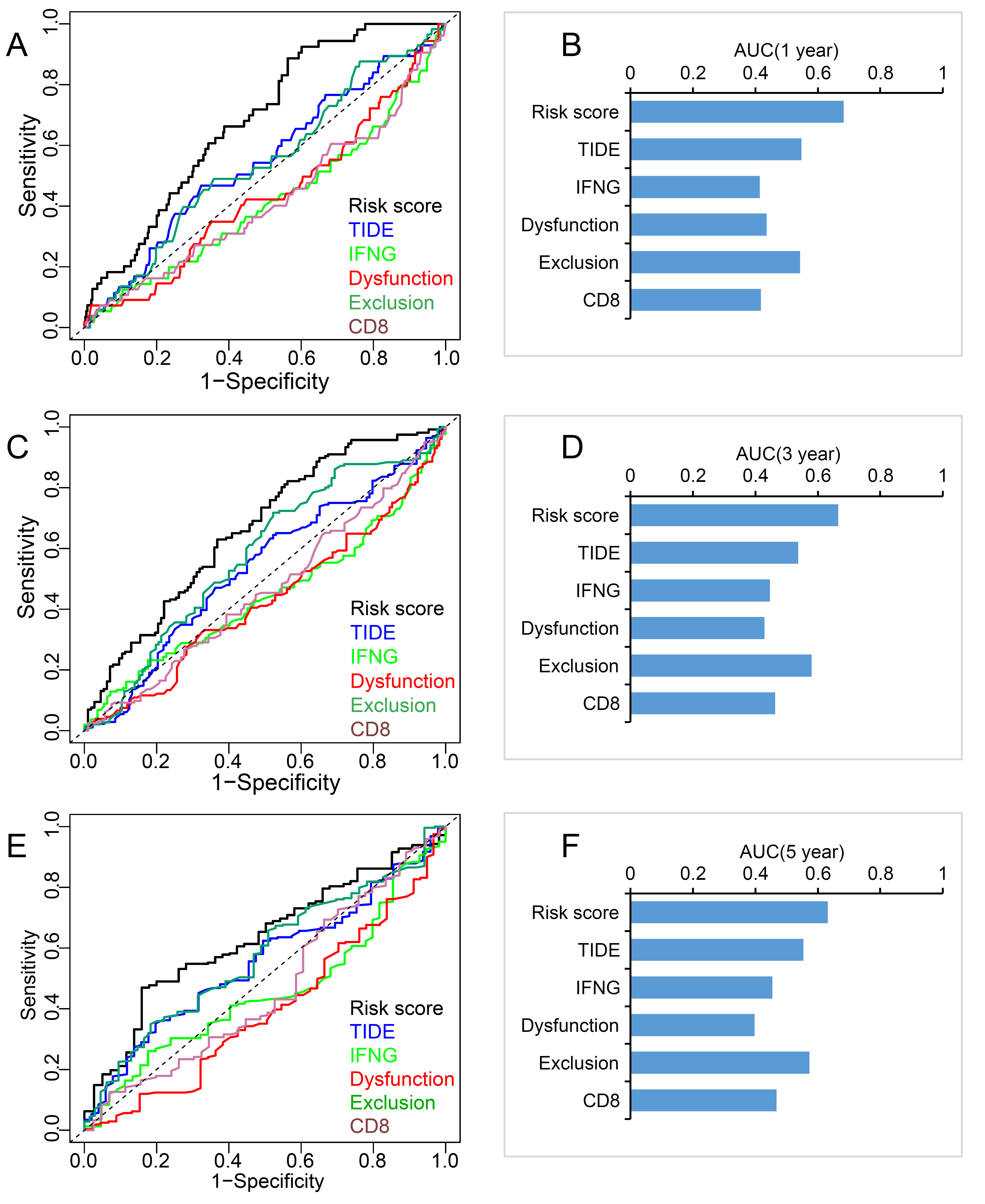


**Figure S6** The comparison of prognosis predict value between TMEM-based signature and TIDE signature. **(A)** ROC curves for the performance of risk score, TIDE prediction score,  IFNG, dysfunction score, exclusion score, and CD8 score in predicting 1 year OS among TCGA cohort. **(B)** The AUC for indicated signatures in predicting 1 year OS among TCGA cohort. **(C)** ROC curves for the performance of risk score, TIDE prediction score,  IFNG, dysfunction score, exclusion score, and CD8 score in predicting 3 year OS among TCGA cohort. **(D)** The AUC for indicated signatures in predicting 3 year OS among TCGA cohort. **(E)** ROC curves for the performance of risk score, TIDE prediction score,  IFNG, dysfunction score, exclusion score, and CD8 score in predicting 5 year OS among TCGA cohort. **(F)** The AUC for indicated signatures in predicting 5 year OS among TCGA cohort.


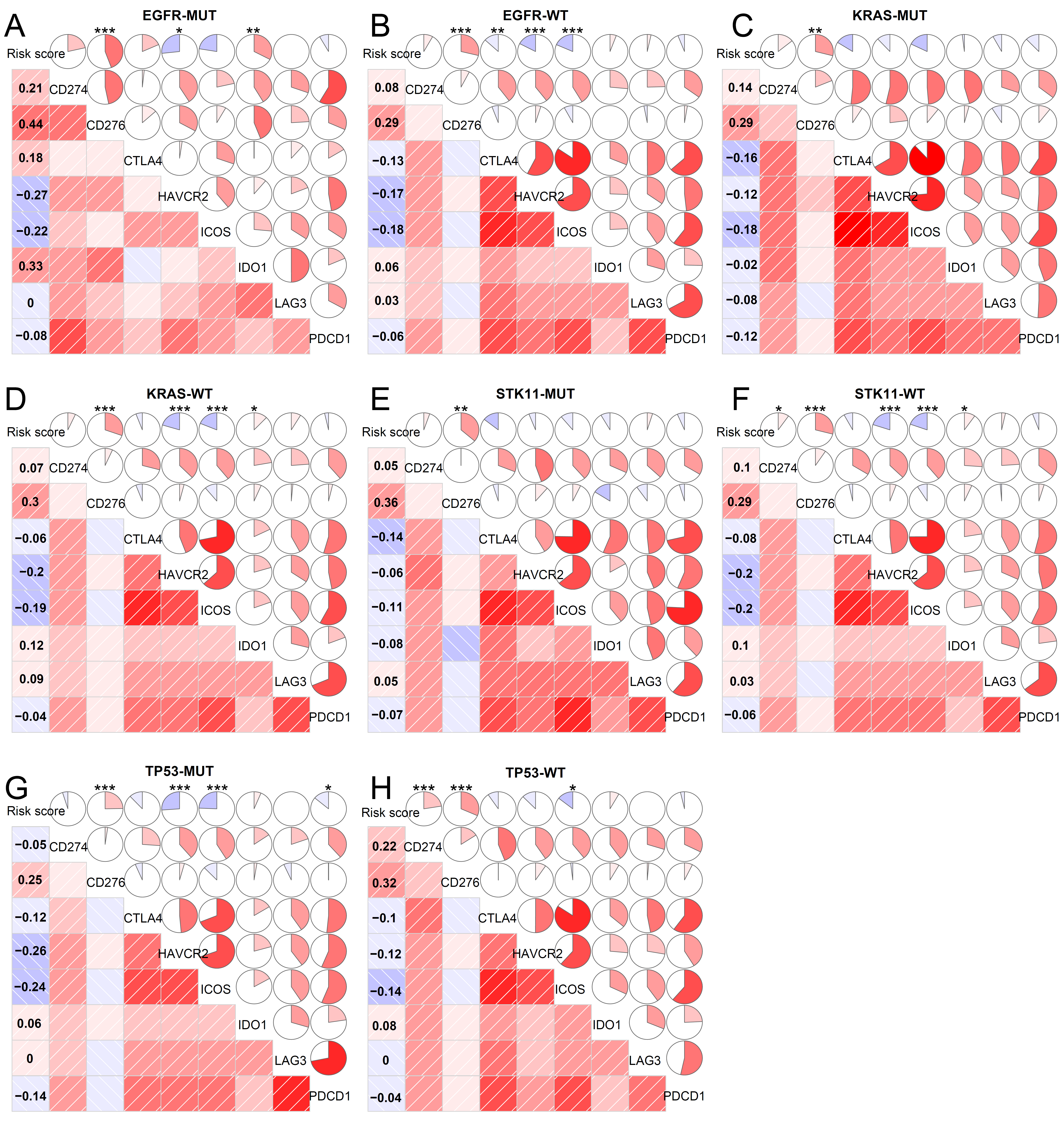


**Figure S7** The correlation heatmap showing the relationship between risk score and immune checkpoints in patients with LUAD of different mutation types. Correlation analysis between risk score and immune checkpoints in patients with EGFR-MUT (**A**), EGFR-WT (**B**), KRAS-MUT (**C**), KRAS-WT (**D**), STK11-MUT (**E**), STK11-WT (**F**), TP53-MUT (**G**), and TP53-WT (**H**). MUT, mutation; WT, wild type. The asterisks represented the statistical p value (**P* < 0.05; ***P* < 0.01; ****P* < 0.001).
